# Plasma metabolomic signatures of breast cancer

**DOI:** 10.3389/fmed.2023.1148542

**Published:** 2023-07-31

**Authors:** Yali Xu, Bin Zhao, Zhu Xu, Xiaogang Li, Qiang Sun

**Affiliations:** ^1^State Key Laboratory of Complex Severe and Rare Diseases, Department of Breast Surgery, Peking Union Medical College Hospital, Chinese Academy of Medical Science and Peking Union Medical College, Beijing, China; ^2^Department of Breast Surgery, The First Affiliated Hospital, Zhejiang University School of Medicine, Hangzhou, China; ^3^Department of Gastrointestinal Surgery II, Renmin Hospital of Wuhan University, Wuhan, Hubei, China; ^4^State Key Laboratory of Complex Severe and Rare Diseases, Department of Medical Research Center, Peking Union Medical College Hospital, Chinese Academy of Medical Science and Peking Union Medical College, Beijing, China

**Keywords:** breast cancer, breast benign abnormalities, metabolomics, lipidomics, biomarker

## Abstract

**Background:**

Breast cancer is a common malignant tumor. A large number of medical evidence shows that breast cancer screening can improve the early diagnosis rate and reduce the mortality rate of breast cancer. In the present study, a wide range of targeted metabolomics profiling was conducted to investigate the plasma signatures of breast cancer.

**Methods:**

A total of 86 patients with benign breast abnormalities (L group) and 143 patients with breast cancer (E group) were recruited. We collected their plasma samples and clinical information. Metabolomic analysis, based on the coverage of a wide range of targeted metabolomics was conducted with ultraperformance liquid chromatography- triple quadrupole-linear ion trap mass spectrometer (UPLC-QTRAP-MS).

**Results:**

We identified 716 metabolites through widely-targeted metabolomics. Serotonergic synapse was the main different metabolic pathway. The fold change of 14 metabolites was considered significantly different (fold change <0.67 or fold change >2; *p* < 0.05). By combining all the 14 metabolites, we achieved differentiation of L group vs. E group (AUC = 0.792, 95%Cl: 0.662–0.809).

**Conclusion:**

This study provided new insights into plasma biomarkers for differential diagnosis of benign abnormalities and breast cancer.

## Introduction

Breast cancer is a common malignant tumor (1). A large number of medical evidence shows that breast cancer screening can improve the early diagnosis rate and reduce the mortality rate of breast cancer (2). Through the analysis of the results of multiple prospective randomized controlled trials (RCTs) and clinical trials, effective screening can reduce the mortality of breast cancer by 20% (2). In addition, due to early occurrence and diagnosis, most tumors are staged in the early stage, and the adverse reactions brought by surgery or chemotherapy are relatively small, so the mortality related to the treatment process of patients is significantly reduced (3–6). For example, when early breast cancer is detected through screening, the probability of breast preservation is higher, the postoperative recovery is faster, the complications are fewer, the appearance of the breast is preserved, and the psychological burden of patients is reduced (7, 8). In addition, the earlier the breast cancer is detected, the smaller the possibility of chemotherapy, and patients can avoid a series of adverse reactions brought on by chemotherapy, such as cardiotoxicity, marrow suppression, and so on. Though imaging is already broadly implemented in breast cancer screening, more convenient plasma markers are still warranted. Metabolome technology has unique advantages in marker discovery and transformation research (9–14).

Here, metabolome was used to compare the difference of serum metabolites between benign and malignant breast abnormalities. We aimed to establish a diagnosis model and evaluate its predictive ability.

## Materials and methods

### Participants

In our research, between 1 November and 31 December 2021, 86 patients with benign breast abnormalities (L group) and 143 participants with breast malignancies (E group) were consecutively enrolled from the Department of Breast Surgery affiliated with Peking Union Medical College Hospital (PUMCH). All patients were from the Han Chinese population. The blood sample collection was conducted according to IFCC/C-RIDL protocols and the study was approved on 2019-4-23 by the institutional committee of PUMCH (ethical number: ZS-1915). Fasting blood samples were taken via venipuncture into Vacuette tubes containing procoagulant, and within 15–30 min after sample collection, the samples were centrifuged at 1,200 × g for 10 min.

### Materials and instrument

Chromatographic grade acetonitrile and methanol were purchased from Merck. Chromatographic grade formic acid, ammonium formate, and ammonia water were obtained from Aladdin. ExionLC AD ultra-Performance liquid chromatography (UPLC) and QTRAP^®^ tandem mass spectrometry (MS/MS) were from AB Sciex (Massachusetts, United States). ACQUITY UPLC HSS T3 C18 column (1.8 μm, 2.1 mm × 100 mm) was purchased from the Waters Corporation (Milford, MA, United States).

### Sample pretreatment

For metabolomics profiling, the plasma sample was prepared by acetonitrile/methanol extraction. In short, 300 μL acetonitrile/methanol containing 20% internal standard solution was vortexed with 50 μL of the sample for 3 min and centrifuged at 12,000 r/min for 10 min at 4°C. Then the supernatant was moved to −20°C for 30 min and centrifuged at 12000 r/min for 3 min at 4°C. The final 180 μL supernatant was used for analysis. The quality control (QC) sample was prepared by mixing the supernatants.

### Gradient elution procedure and ESI source conditions for metabolomic profiling

An LC-ESI-MS/MS system (UPLC, ExionLC AD^1^; MS, QTRAP^®^ System^2^) was used to analyze the sample extracts. ACQUITY UPLC HSS T3 C18 column (1.8 μm, 2.1 mm × 100 mm, Waters Corporation, Milford, MA, United States) was used for separation. The flow rate was 0.4 mL/min, the column temperature was set as 40°C, and the injection volume was set as 2 μL. Mobile phase A contained 0.1% formic acid in water. Mobile phase B was acetonitrile containing 0.1% formic acid. The gradient elution procedure was as follows: mobile phase A was 95% for 10 min and linearly reduced to 10% in 11 min, then rise to 95% in 13 min.

A triple quadrupole-linear ion trap mass spectrometer (QTRAP), QTRAP^®^ LC-MS/MS System, combined with an ESI Turbo Ion-Spray interface was used for MS data acquiring. The electrospray ionization (ESI) source temperature was 500°C, mass spectrometry voltage was −4,500 V (negative) and 5,500 V (positive), ion source gas1 (GS I) was 55 psi, ion source gas2 (Gas2) was 60 psi, and curtain gas (CUR) was 25 psi, high collision-activated dissociation (CAD) parameter. Each ion pair in the triple quadrupole (Qtrap) was scanned and detected in accordance with their optimized declustering potential (DP) and collision energy (CE).

### Mass spectrometry data processing and statistical analysis

Analyst 1.6.3 software was used to process the metabolomics raw MS data. Based on the self-established targeted standard database (MWDB), the information and secondary spectral data were qualitatively analyzed based on retention time, parent and daughter ions of detected substances. Multivariate statistical analysis was conducted with the R package and MetaboAnalyst 5.0 (Xia Lab @ McGill Sweden). *p* < 0.05 was considered statistically significant.

## Results

### Clinical information of patients

Clinical characteristics of study participants with benign (*n* = 86) and malignant (*n* = 143) abnormalities are shown in Table 1. The mean age of participants was 44.43 ± 1.62 years and 54.95 ± 13.48 years for the L and E groups, respectively. Fibroadenoma was the main type of benign breast abnormality. Of the malignancies, 77.62% (111/143) were ductal carcinoma and 64.79% (92/143)were early-stage breast cancers (stage 0 and I). A total of 83.33% (115/138) of breast cancers were luminal, while Her-2 overexpression and triple-negative subtypes accounted for 9.79 and 6.29%, respectively.

**Table 1:** Clinical characteristics of study participants with benign and malignant abnormalities.

		Malignant (*n* = 143)	Benign (*n* = 86)	*p* value
Age (years)mean ± SD		54.95 ± 13.48	44.43 ± 1.62	<0.00001
BMI (mean ± SD, kg/m^2^)		23.98 ± 3.15	23.02 ± 3.22	0.03
Menopause	Pre-menopausal	34 (23.78%)	41 (47.67%)	<0.00001
	Peri-menopausal	29 (20.28%)	22 (25.58%)	
	Post-menopausal	79 (55.24%)	23 (26.74%)	
Cancer type	Ductal	111 (77.62%)	–	–
	Lobular	4 (2.80%)	–	
	Ductal and lobular	2 (1.40%)	–	
	Other	26 (18.18%)	–	
TNM stage	0	14 (9.79%)	–	–
	I	78 (54.55%)	–	
	II	30 (20.98%)	–	
	III	20 (13.99%)	–	
	Unknown	1 (0.70%)		
ER status	Negative	26 (18.18%)	–	–
	Positive	112 (78.32%)	–	
	Unknown	5 (3.50%)	–	
PR status	Negative	35 (24.48%)	–	–
	Positive	102 (71.33%)	–	
	Unknown	6 (4.20%)	–	
Her-2 status	Negative	25 (17.48%)	–	–
	Positive	112 (78.32%)	–	
	Unknown	6 (4.20%)	–	
Molecular subtypes	Luminal A	41 (28.67%)	–	–
	Luminal B1	25 (17.48%)	–	
	Luminal B2	49 (34.27%)	–	
	Her-2 overexpression	14 (9.79%)	–	
	Triple-negative BC	9 (6.29%)	–	
	Unknown	5 (3.50%)		

BMI, body mass index; TNM, tumor, nodes, metastases; ER, estrogen receptor; PR, progesterone receptor; and Her-2, human epidermal growth factor receptor-2.

### Metabolomics profiling of plasma

**Figure 1:**
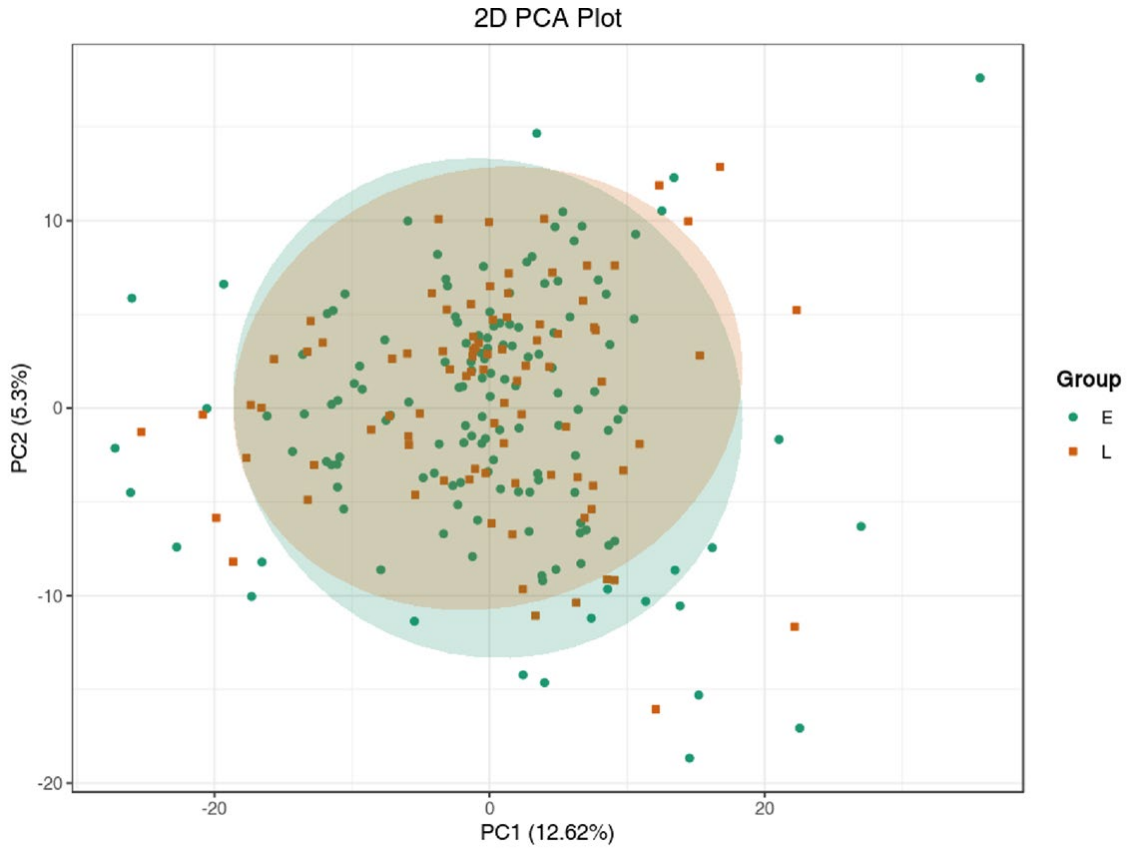
PCA model of metabolomics for benign abnormality (L group) and malignant abnormality (E group).

Unsupervised PCA (principal component analysis) was conducted to preliminarily characterize the metabolite differences of each group and the variation degree within the groups. The unsupervised PCA was conducted after the data unit variance scale. PCA results showed a little separation trend of metabolome among groups (Figure 1).

**Figure 2:**
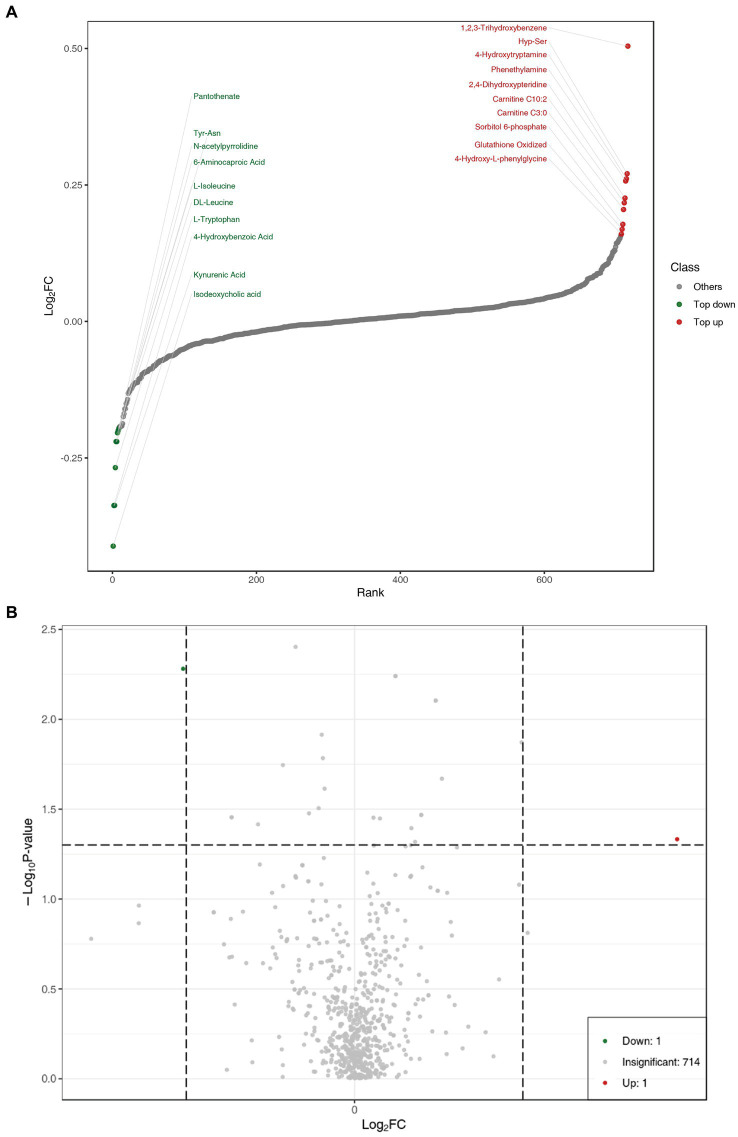
**(A)** Dynamic distribution of metabolite content differences. **(B)** Volcano plot of metabolomics for benign abnormality (L group) and malignant abnormality (E group).

In order to show the overall metabolic difference more clearly and intuitively, the metabolites in the comparison group were calculated by the value of fold change (FC). After calculation, according to the value of FC, we determined the dynamic distribution of metabolite content, and the top 10 metabolites upregulated and downregulated were labeled (Figure 2A). The top 10 upregulated metabolites were 1,2,3-trihydroxybenzene, hyp-ser, 4-hydroxytryptamine, phenethylamine, 2,4-dihydroxypteridine, carnitine C10:0, carnitine C3:0, sorbitol 6-phosphate, glutathione oxidized, and 4-hydroxy-L-phenylglycine, whereas the top 10 downregulated metabolites were pantothenate, tyr-asn, N-acetylpyrrolidine,6-aminocaproic acid, L-Isoleucine, DL-leucine, L-tryptophan, 4-hydroxybenzoic acid, kynurenic acid, and isodeoxycholic acid (Table 2).

**Table 2:** Significant metabolites for comparison of participants with benign and malignant abnormalities.

Q1 (m/z)	Metabolite	Formula	Benign (L group) */* Malignant (E group)			
			CAS	*p*-value	Fold change	Type
114.1	N-acetylpyrrolidine	C6H11NO	4030-18-6	1.290E-01	8.746E-01	Down
122.1	Phenethylamine	C8H11N	64-04-0	8.326E-02	1.195E+00	Up
132.1	L-Isoleucine	C6H13NO2	73-32-5	1.787E-01	8.681E-01	Down
132.1	6-Aminocaproic Acid	C6H13NO2	60-32-2	1.186E-01	8.585E-01	Down
132.1	DL-Leucine	C6H13NO2	328-39-2	1.186E-01	8.585E-01	Down
137.0	4-Hydroxybenzoic Acid	C7H6O3	99-96-7	1.364E-01	7.916E-01	Down
165.3	2,4-Dihydroxypteridine	C6H4N4O2	487-21-8	2.803E-01	1.170E+00	Up
177.1	4-Hydroxytryptamine	C10H12N2O	570-14-9	1.343E-02	1.199E+00	Up
188.0	Kynurenic Acid	C10H7NO3	492-27-3	1.088E-01	7.917E-01	Down
205.1	L-Tryptophan	C11H12N2O2	73-22-3	5.234E-03	8.306E-01	Down
206.1	N-Acetyl-L-phenylalanine	C11H13NO3	2018-61-3	4.084E-01	1.007E+00	Up
218.1	Carnitine C3:0	C10H19NO4	17298-37-2	5.516E-01	1.153E+00	Up
218.1	Pantothenate	C9H17NO5	79-83-4	8.921E-01	8.708E-01	Down
219.1	hyp-ser	C8H14N2O5	–	1.544E-01	1.206E+00	Up
296.1	Tyr-Asn	C13H17N3O5	151145-11-8	2.113E-01	8.732E-01	Down
316.2	Carnitine C10:0	C17H33NO4	–	6.081E-01	1.020E+00	Up
391.3	Isodeoxycholic acid	C24H40O4	566-17-6	1.665E-01	7.518E-01	Down
523.1	Sorbitol 6-phosphate	C6H15O9P	20479-58-7	5.135E-01	1.131E+00	Up
611.1	Glutathione Oxidized	C20H32N6O12S2	121-24-4	6.782E-01	1.124E+00	Up

Univariate statistical analysis (parameter test and non-parameter test) and multivariate statistical analysis (principal component analysis and partial least square discriminant analysis) should be combined to excavate differential metabolites. Variable importance in projection (VIP) of the OPLS-DA model and *p*-value (Wilcoxon rank-sum test) or fold change (FC) were used to select differential metabolites. The volcano plot was mainly used to demonstrate the relative content difference of metabolites between the L and E groups (Figure 2B).

### Hierarchical cluster analysis

**Figure 3:**
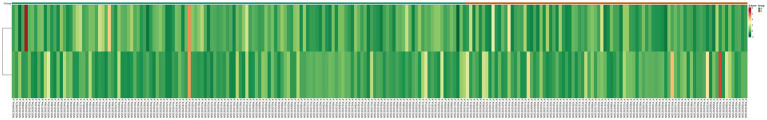
Heatmap of different features for benign (L group) and malignant abnormalities (E group).

Cluster analysis is a method for the classification of multivariate statistical analysis according to samples’ characteristics. In order to observe the relative content of metabolites, we used unit variance (UV) treatment for the original relative content of the different metabolites identified by the screening criteria and drew heat maps with the R software package (Figure 3). As Figure 3 shows, some metabolites were different among different groups. This result is consistent with the PCA and volcano plot analysis.

### KEGG and regulatory network analysis

**Figure 4:**
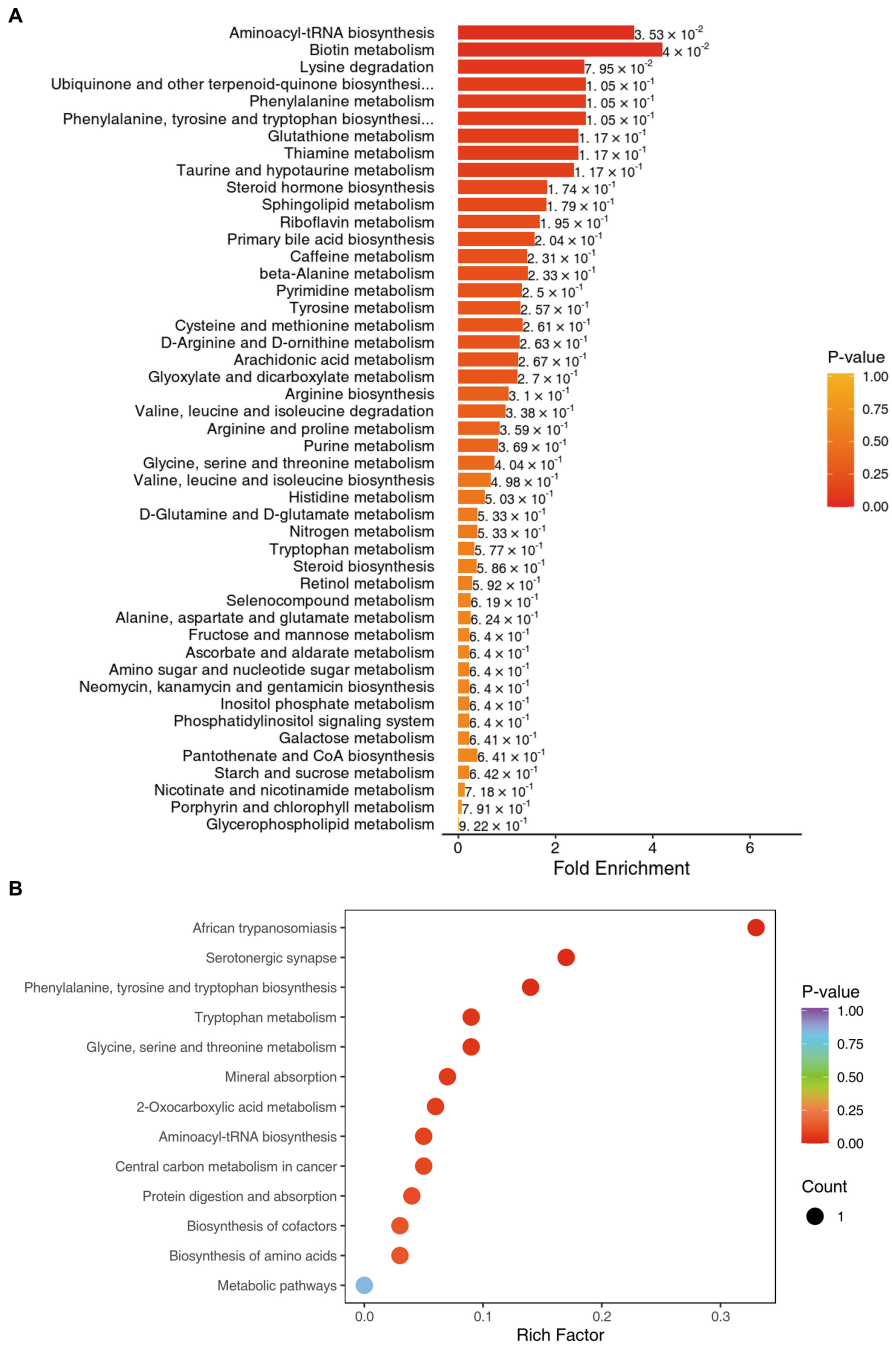
**(A)** KEGG MSEA enrichment analysis diagram for benign (L group) and malignant abnormalities (E group). **(B)** KEGG enrichment map of differential metabolites for benign (L group) and malignant abnormalities (E group).

**Figure 5:**
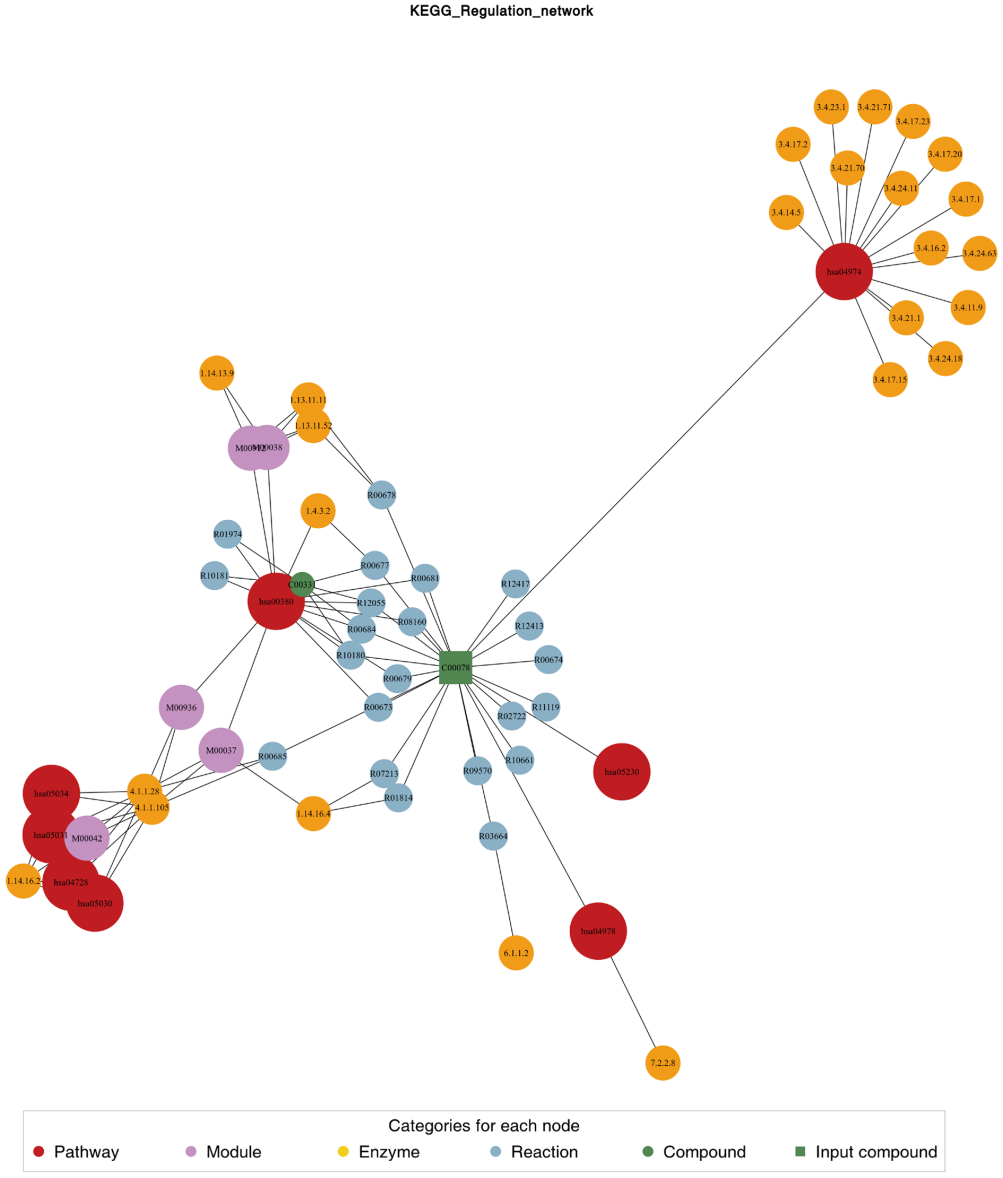
Differential metabolite regulatory network.

We used the KEGG compound database^3^ to annotate identified metabolites. After annotation, we mapped the metabolites using the KEGG Pathway database.^4^ Metabolite sets enrichment analysis (MSEA) was used to analyze the significantly regulated pathways (Figure 4). African trypanosomiasis and serotonergic synapse were the main different metabolic pathways. We also conducted regulatory interaction network analysis according to the KEGG database, which was displayed by network plot (Figure 5).

### Biomarker evaluation

**Figure 6:**
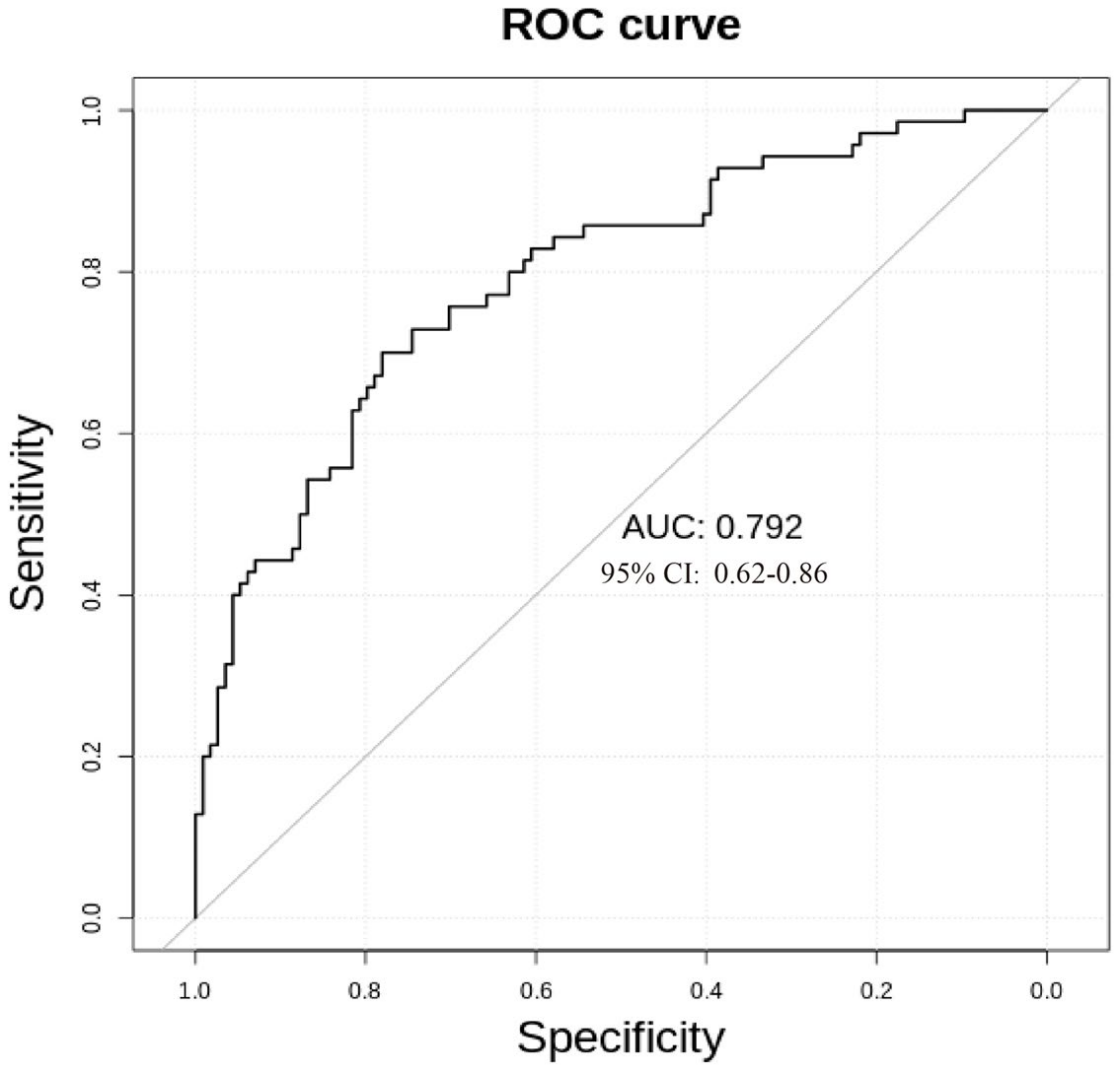
ROC curve for benign (L group) and malignant abnormalities (E group).

We used receiver operating characteristic (ROC) analysis to evaluate the significantly different metabolites. Multivariate exploratory ROC analysis was performed using MetaboAnalyst 5.0. The differentiation of L group and E group could be reached by combining 14 metabolites (AUC = 0.792, 95%Cl: 0.662–0.809) (Figure 6).

## Discussion

Discrimination of breast abnormalities between benign and malignant before surgery has been a challenging issue in clinical practice, though imaging has shown advantages in detecting breast abnormalities. The inpatients with various imaging abnormalities were admitted for surgeries. The profiling of peripheral plasma targeted metabolomics could supplement further information to imaging. This wide range of untargeted metabolomics profiling showed that serotonergic synapse was the main different metabolic pathway. The dynamic distribution of metabolite content difference was drawn according to the value of FC, and the top 10 metabolites upregulated and downregulated were labeled, as shown in Figure 2. In Figure 3, the x-coordinate represents the logarithm value (log_2_FC) of the multiple of the relative content difference of a certain metabolite, the difference significance level (−log_10_*p*-value). Figure 3 was drawn under the FC and *p*-value double screening condition. The top 10 upregulated metabolites (1,2,3-trihydroxybenzene, hyp-ser, 4-hydroxytryptamine, phenethylamine, 2,4-dihydroxypteridine, carnitine C10:0, carnitine C3:0, sorbitol 6-phosphate, glutathione oxidized, and 4-hydroxy-L-phenylglycine) and top 10 downregulated metabolites (Pantothenate, tyr-asn, N-acetylpyrrolidine,6-aminocaproic acid, L-Isoleucine, DL-leucine, L-tryptophan, 4-hydroxybenzoic acid, kynurenic acid, and isodeoxycholic acid) could be applied in inpatients whose breast abnormalities are difficult to distinguish between benign and malignant.

The technique of mass spectrometry has quickly developed. By identification of metabolic biomarkers indicative of various pathologies, new MS techniques could further our understanding of diseases. In our study, we acquired metabolite data by QTRAP^®^ LC-MS/MS System combined with an ESI Turbo Ion-Spray interface, which could explore a better panel of metabolites for the classification of breast abnormalities. We formed a panel of 14 significantly different metabolites (*p* < 0.05; fold change >2 or fold change <0.67), which might be used more conveniently in clinical practice (AUC = 0.792, 95%Cl: 0.662–0.809). Metabolic pathway analysis showed serotonergic synapse was the main different metabolic pathway, which could add new knowledge in metabolites studies (15, 16). Together with the signaling pathway of HIF-1 and ferroptosis, serotonergic synapse-related DEGs were usually significantly enriched (17). The serotonergic synapse signal pathway was activated by the core gene protein kinase PKA and the expression of 5-HT and GABA was increased, hence insomnia symptoms were improved and anxiety was alleviated (18).

Serotonin is involved in a variety of physiological processes, such as platelet activation, liver regeneration, pancreatic cell function, regulation of vasoconstriction, and repair after ischemic injury. In addition, serotonin acts as an important inflammatory mediator, affecting immune functions such as leukocyte adhesion and migration. Serotonin is also associated with cell proliferation and cancer, promoting the growth of hepatocellular carcinoma. Serotonin levels also play a role in the Warburg effect of pancreatic cancer, with serotonin and serotonin receptors possibly modulating the phenotype and function of various immune cells (19, 20). This may be linked to and associated with patient emotion, but this needs to be further studied.

Xie et al. (21) found that serotonin expression was also much higher in triple-negative (PR-, ER-, HER-2) breast cancer (TNBC) and triple-positive breast cancer (TPBC) compared to para-carcinoma tissues (PCTs). However, we did not find this relationship, and we concluded that this may be related to the sample source since we used peripheral blood, whereas Xie et al. (21) used tissue and cell lines.

As to matrix metalloproteinases (MMPs), no difference between the benign and malignant breast abnormalities was observed in our study. By now the biological function of MMPs in cancer remains controversial. Originally, MMPs are proteases capable of remodeling the extracellular matrix, but they have been demonstrated to play numerous additional biologic roles in inflammatory, autoimmune, cancer, and pathogen-mediated diseases (22). As MMPs are the most prominent proteinases involved in tumorigenesis, hence they might be potential therapeutic targets in breast cancer (23). There are thousands of MMPs suggested substrates and only a few hundred have been validated (24). In disease progression and resolution, the contributions of MMPs could be both beneficial and detrimental. They were initially recognized to promote tumor progression by remodeling the extracellular matrix through their proteolytic activity. Afterward, it was revealed that the same MMP can exert opposing roles depending on the cell type in which it is expressed or the stage of cancer. Hence, the role of MMPs’ double edge sword in tumorigenesis and progression leads to no difference between benign and malignant breast abnormalities and warrants further research.

Discrepancies do exist between our study and previous results (16, 25–27), which is a common phenomenon in similar studies. Some of the results might even suggest the opposing direction, which warrants more work to further our understanding of tumors. By now we know more about tumors by different techniques (especially MS), while different study results could not be systemically summarized. A larger sample size might help screen out optimal panels, but might also lead to more complicated information. In this project, we used a wide range of targeted metabolomics methods and used standard molecule data as a control. We were able to determine the absolute content of key molecules. Furthermore, we are enlarging the number of patients and trying to develop a method of detecting the key molecules in this study, which will make the results more robust. More work should be done on data processing and analyzing so as to obtain genuine and useful explanations.

Though our study adds further information to peripheral plasma profiling of untargeted metabolomics, there are some limitations. First, no information on plasma metabolomics of healthy controls was screened, hence the results could only be adapted to clinical practice. Further study on healthy women should be done in order to adapt to population screening of breast abnormalities. Second, the sample size was relatively small. An enlarged sample size would be helpful to further confirm the profiling results. Third, the mechanism of significantly different metabolites was unclear and further study is warranted.

In conclusion, our study revealed the metabolic heterogeneity between benign breast abnormalities and breast cancers, and a panel of 14 metabolites was screened out to assist in the differentiation of benign and malignant breast abnormalities for inpatients in clinical settings. Since we did not find a single excellent specific biomarker for the differentiation of benign and malignant breast abnormalities, we combined many metabolites and evaluated them by AUC rank. However, this model needs to be validated in other patients and other centers. Combined with imaging, the detection of metabolites by MS might improve clinical diagnosis accuracy and partly relieve the anxiety of some inpatients regarding breast abnormalities.

## Data availability statement

The raw data supporting the conclusions of this article will be made available by the authors, without undue reservation.

## Ethics statement

The studies involving human participants were reviewed and approved by institutional committee of PUMCH. The patients/participants provided their written informed consent to participate in this study.

## Author contributions

YX and XL: conception and design. XL and QS: administrative support. BZ, ZX, and XL: provision of study materials or patients. XL and YX: collection and assembly of data. XL and ZX: data analysis and interpretation. YX, BZ, ZX, XL, and QS: manuscript writing. All authors contributed to the article and approved the submitted version.

## Funding

This work was supported by the CAMS Initiative for Innovative Medicine (2021-I2M-1-014) and the National High Level Hospital Clinical Research Funding (2022-PUMCH-A-263).

## Conflict of interest

The authors declare that the research was conducted in the absence of any commercial or financial relationships that could be construed as a potential conflict of interest.

## Publisher’s note

All claims expressed in this article are solely those of the authors and do not necessarily represent those of their affiliated organizations, or those of the publisher, the editors and the reviewers. Any product that may be evaluated in this article, or claim that may be made by its manufacturer, is not guaranteed or endorsed by the publisher.

## Footnotes

^1^https://sciex.com.cn/

^2^https://sciex.com/

^3^http://www.kegg.jp/kegg/compound/

^4^http://www.kegg.jp/kegg/pathway.html
